# Failure Analysis of TEVG’s I: Overcoming the Initial Stages of Blood Material Interaction and Stabilization of the Immune Response

**DOI:** 10.3390/cells10113140

**Published:** 2021-11-12

**Authors:** Maria A. Rodriguez-Soto, Natalia Suarez Vargas, Alejandra Riveros, Carolina Muñoz Camargo, Juan C. Cruz, Nestor Sandoval, Juan C. Briceño

**Affiliations:** 1 Department of Biomedical Engineering, Universidad de los Andes, Bogotá 111711, Colombia; na.suarez122@uniandes.edu.co (N.S.V.); ra.riveros11@uniandes.edu.co (A.R.); c.munoz2016@uniandes.edu.co (C.M.C.); jc.cruz@uniandes.edu.co (J.C.C.); 2 Department of Congenital Heart Disease and Cardiovascular Surgery, Fundación Cardio Infantil Instituto de Cardiología, Bogotá 111711, Colombia; nsandoval@cardioinfantil.org; 3 Department of Research, Fundación Cardio Infantil Instituto de Cardiología, Bogotá 111711, Colombia

**Keywords:** tissue-engineered vascular grafts, regeneration, failure, immune response

## Abstract

Vascular grafts (VG) are medical devices intended to replace the function of a diseased vessel. Current approaches use non-biodegradable materials that struggle to maintain patency under complex hemodynamic conditions. Even with the current advances in tissue engineering and regenerative medicine with the tissue engineered vascular grafts (TEVGs), the cellular response is not yet close to mimicking the biological function of native vessels, and the understanding of the interactions between cells from the blood and the vascular wall with the material in operative conditions is much needed. These interactions change over time after the implantation of the graft. Here we aim to analyze the current knowledge in bio-molecular interactions between blood components, cells and materials that lead either to an early failure or to the stabilization of the vascular graft before the wall regeneration begins.

## 1. Introduction

During the last decade, cardiovascular diseases caused more than 30% of the deaths worldwide. Most of them exhibit an altered structure of the blood vessels supplying the heart, brain, and limbs. When the blood flow is sufficiently impaired, the lack of nutrients and metabolite perfusion leads to permanent damage to the target tissue. The existing strategies for revascularization include the use of vascular grafts (VG). VG are medical devices intended to replace or reconnect a blood vessel. The most popular synthetic VGs in the market are made from Polytetrafluoroethylene (PTFE—also known as Teflon^®^) and Polyethylene terephthalate (PET—also known as Dacron^®^). However, despite being accessible and useful, their patency rates when implanted on vessels with diameters below 6 mm or under complex hemodynamic conditions with reduced blood flow (such as venous grafts) are low, with only 32% success after 2 years. They consequently fail under different circumstances associated with their non-regenerative properties, leading to chronic inflammatory responses that will ultimately affect the VG’s structural integrity and function [1,2]. For that reason, re-interventions are generally needed [3,4], and the selection of the appropriate treatment is challenging.

Tissue engineering and regenerative medicine provide an alternative route to overcome the limitations of synthetic non-degradable VGs. Tissue-engineered vascular grafts (TEVGs) aim to have the ability to remodel, grow, and repair the vascular wall upon implantation for a more hemodynamic-responsive conduit, able to maintain patency. Nevertheless, the design of TEVGs is challenging given the multiple parameters involved in a full description of the blood–material interactions [5,6].

**Figure 1:**
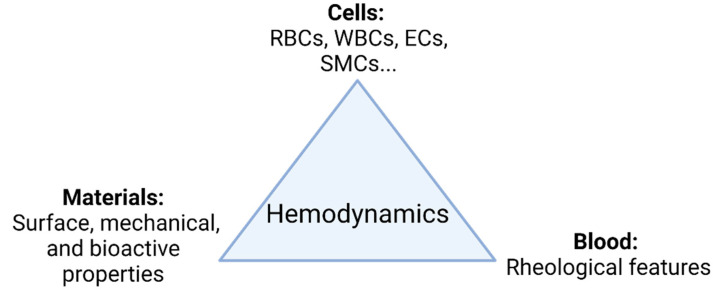
TEVG triad. Regeneration is mainly governed by the interplay of involved cells, biomaterials, and the microenvironment. Changes in the hemodynamic context directly impact how the interplay of the parameters takes place and ultimately the performance of the TEVG under functional physiological conditions (Created with BioRender.com, accessed on 7 November 2021).

The main goal in the development of TEVGs is to recover the physiological function of the native vessel through the formation of new functional tissue able to respond adequately to hemodynamics. In general, as shown in Figure 1, there are three elements interacting during the vascular wall regeneration: cells, biomaterials, and the local microenvironment providing physical and chemical stimulation signals, including the TEVG hemodynamics.

Nevertheless, although the pathophysiology of the failure causes of different kinds of VGs has been previously reported, there is still a gap in the literature regarding the strategies to overcome the failure of TEVGs, because the biomolecular mechanisms of regeneration in the vascular wall are still under study due to their multifactorial origin [7]. According to a considerable number of reports, the early failure of a VG is defined to occur within the first weeks after implantation. The current knowledge suggests that the possible reasons for patency loss and the subsequent failure of VGs and TEVGs is the development of pro-coagulant and inflammatory phenotypes of the interacting blood cells and the cells in the vascular wall near the implantation site [8,9].

In this way, the success of a TEVG depends on the result of the complex interactions of the biomaterial with the cells from the blood, immune system, and stem cells, as well as the blood and the surrounding vessels under the hemodynamic stimulus provided by the blood flow [10]. As an attempt to provide detailed information for the rational design of a successful TEVG that would not undergo any of these failure events, this review aims to provide a comprehensive description of the biomolecular interactions between the blood components, the perivascular cells, and the material in the hemodynamic context of VG. To this end, we carried out a detailed analysis of pre-clinical in vivo studies in which we identified the common events related to success or patency loss. Then, we put forward sound hypotheses in light of a mechanistic description of the interactions between cells, blood, and biomaterials under hemodynamic conditions that must occur in case of success, as well as the ones that will promote failure.

## 2. Materials and Methods

To identify the causes of failure of vascular grafts in in vivo environments, we critically analyzed the review by Skovrind, Harvald, Belling, Damsgaard Jorgensen, Lindholt, and Andresen, where they conducted a meta-analysis of a large database of in vivo studies. We further included more recent studies, analyzed the events related to success and failure, and classified them according to the timing and the impact on the graft permeability. A total of 49 studies were reviewed, and the information regarding the TEVGs and their controls was used to compare TEVGs and other kinds of VGs. We then classified them according to the animal model and compared the studies including preseeded TEVGs and non-preseeded TEVGs. We analyzed the maturation times of these vascular grafts and rationalized the identified failure causes considering the interactions between the cells, the blood extracellular matrix (bECM), and the materials under the specific hemodynamic conditions of a VG.

The search for documents was carried out in PubMED, Access Medicine, Annual Reviews, EBSCO, EMBASE, Nature Journals, Science Direct, SCOPUS, Springer, Springerlink, and Wiley Online Library. The used keywords were “vascular grafts”, “design”, “cell behavior”, “blood components”, “biomimetic”, “Biomaterials”, “failure”, “approaches”. The Boolean operator AND was the connector for the search. Only documents published in English were considered. Only articles in which the main body contents and/or abstract accounted for the detailed cell behavior and vascular graft under the physiological and pathological conditions induced by the graft material were selected. The information was classified considering the found differences between the current VGs and compared with the TEVG responses. The collected documents were sorted out according to the relevance of the development and the stage of testing. Then, the information was further organized by looking at the interactions between cells and TEVGs.

## 3. Phases of the Vascular Graft Response

If patency loss occurs within the first days or weeks, it is usually induced by thrombosis, that might be caused mainly by the biomaterial properties (i.e., thrombogenic protein adsorption due to negatively charged surfaces, hemolytic surfaces, and immunogenic surfaces) [1] or due to an infection caused by intraoperative bacterial contamination or the spread of an infection from a nearby area [2,11].

**Figure 2:**
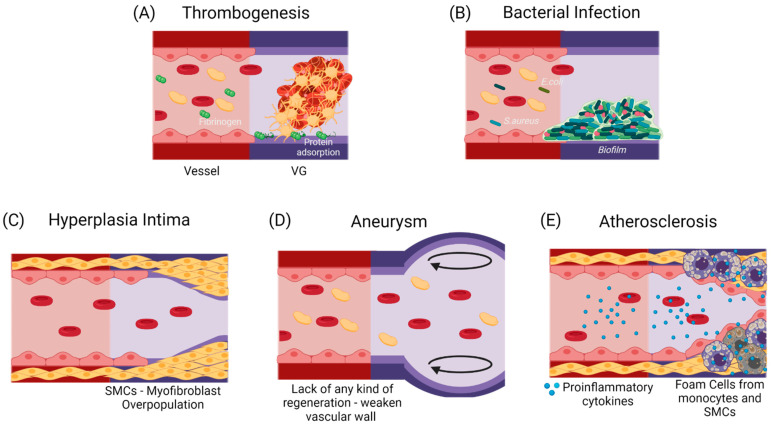
Commonly reported causes for TEVGs’ failure. (**A**) An early failure has been reported to be associated with the inherent thrombogenicity of the biomaterial due to protein adsorption and platelet activation [16]. (**B**) An early failure can also be associated with infection, in which a formed biofilm impairs treatment and induces inflammation [17]. Due to the regulatory mechanisms of endothelium, Mid-Late failure can be mostly related to an incomplete endothelialization. (**C**) an incomplete endothelium and a mismatch in the mechanical properties with a flow disturbance will induce the activation of a synthetic phenotype in SMCs, that will overproliferate and decrease the vessel lumen [17]. (**D**) The lack in the mechanical properties of the TEVG required for specific hemodynamic conditions or a faster biodegradability rate than the vascular wall regeneration is expected to cause aneurysms [18,19]. (**E**) A slower biodegradability rate than the vascular wall regeneration is likely to cause a higher influx of pro-inflammatory cells, that might lead to a dysregulated balance between the synthesis and the degradation of the novo extracellular matrix, and if there is a chronic inflammatory stimulus, atherogenesis can occur with calcium and phosphate deposition in the disorganized tissue [20]. While thrombogenesis and hyperplasia intima have been reported mainly on small-diameter TEVGs, and bacterial infection and aneurysms are typical in larger TEVGs, atherosclerosis has been shown in both types of TEVGs [17]. (Created with BioRender.com, accessed on 7 November 2021).

If the failure occurs anytime between the first months and the first two years after implantation, it has been shown to be mostly related to a compensation mechanism. In the first case, it might lead to hyperplasia intima, in which the smooth muscle cells and myofibroblasts overproliferate, or due to aneurysm generation, in which the mechanical properties of the vascular wall are inadequate to support the blood flow pressures [12,13]. At a longer term, atherosclerosis or vascular wall calcification might occur, in which a chronic inflammatory process will modify the local calcium metabolism, stenosing and hardening the vascular wall [14,15]. Figure 2 shows the possible failure causes in TEVGs. In most of the cases, the ultimate result is the alteration of the vessel lumen, compromising the blood flow, which can also be life-threatening. Even if there are interventions such as catheter-assisted thrombolysis, the placement of a stent, or the surgical revision of the graft to recover the blood flow, VG tend to fail over time [1,2].

Here, we studied the failure causes considering the in vivo studies (in different animal models) reported by Skovrind et al. [19] and including some more recent studies in rabbits, pigs, and dogs. Then, we classified them according to the moment at which graft fails after implantation and the pathophysiology that leads to patency loss in the graft in light of the TEVG triad and the impact of such reduction in perfusion. Considering the general stages of integration of a vascular graft and the expected remodeling process, we have divided the integration process of a TEVG in three phases, defined arbitrarily by our research group: Phase (I): Peri-implantation period, in which success is defined by the capacity of the graft to overcome the surgery. Every graft that overcomes this stage is expected to begin a reendothelialization process. Phase (II) Period after the implantation and the inflammatory response, in which success is defined by the absence of indications of chronic inflammation and the reendothelialization of the graft. If the graft overcomes this phase and forms a proendothelial lining, it will expectedly begin remodeling the vascular wall. Phase (III) Vascular wall remodeling and device maturation, in which success is defined by the graft acting as an adequate substrate for repopulating and modulating smooth muscle cells, the endothelial lining, and the external fibrous layer in a controlled manner. Then, we described possible failure causes that could occur within the time periods limited by the phases defined previously.

During the first 24 h in phase I, a foreign body response and inflammatory and hemostatic processes dominate the interactions between the TEVG, the cells, and the blood components at the implantation site. Phase II lies between the first weeks to the first months, and in this case, changes and the adaptation to the hemodynamic behavior at the implantation site determine the TEVG integration of the adjacent tissue. Lastly, phase III occurs when the VG starts to function for its intended application and remodels tissue in response to the governing hemodynamics and cell turnover.

Following the Skovrind et al. [19] study, we analyzed five types of models (caprine, swine, dog, rabbit, and rat), which were contained in a total of 119 studies. (Table 1).

Table 2 shows the preclinical animal studies used for the survival curve of VG and the identifications of the phases in the regeneration of TEVGs as well as their failure causes. The data regarding controls and commercial VGs was also used. PBMC (peripheral blood mononuclear cells), EC (endothelial cells), SMCs (Smooth muscle cells), BMMNCs (bone marrow mononucleated Cells), MVEC (mammary vascular endothelial cells), HIAECs (primary human iliac artery endothelial cells), HOB (human osteoblasts), PLA (polylacticacid), PCL (polycaprolactone), PGA (polyglycolicacid), PU (polyurethane), PVA (polyvinyl acetate), PS (polystyrene). The asterisk next to the year (*) in the author column indicates the additional studies included apart from the ones reported by Skovrind et al [19].

**Figure 3:**
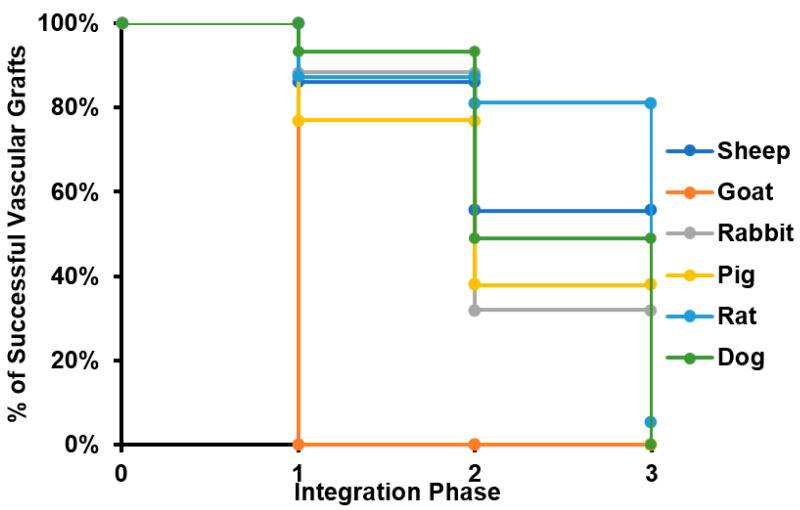
Survival curve of tissue-engineered vascular grafts in preclinical studies. The survival curve presented starts with all the specimens participating in preclinical studies for each of the species evaluated. When a given number of individuals does not overcome a given phase of the integration process, it is subtracted from the total number of specimens participating, reducing the percentage of animals surviving the study.

Considering that all specimens evaluated for caprine model lost patency within the peri-implantation period, this model was disregarded for further analyses. The results suggest that most of the failure cases are related to the transition from phase II to phase III, with an average 14% of grafts failing in early stages and 35% of them reporting loss of patency or the need for removal before the beginning of the remodeling process. This strongly indicates that most of the failure cases are related to inflammatory responses. Because an important number of studies ended suddenly before failure (mainly due to the impairment of the proper blood flow), it was impossible to determine the remodeling performance of such grafts. In these cases, we classified the graft within the defined phases and considered the explanting conditions in terms of level of vascular wall remodeling, reendothelialization, presence of thrombus, and general integrity. We calculated the percentage of successful VGs for each of the phases to build the survival curve shown in Figure 3.

**Figure 4:**
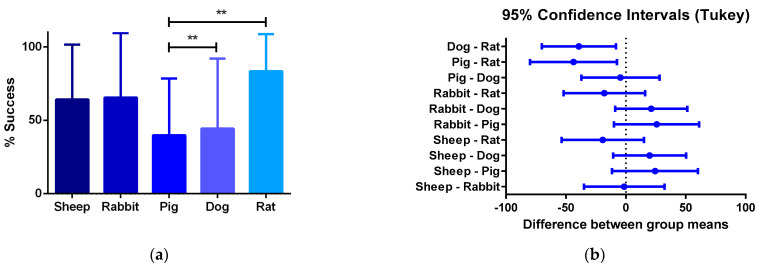
(**a**) One-way ANOVA analysis of different success percentages of preclinical studies in different species: Success is defined by the capacity of the graft to overcome the stabilization of the immune response and start the remodeling process of the vascular wall. From all the specimens participating in pre-clinical trials, the number of individuals participating in the preclinical studies and the percentage of the total of individuals from each species that is successful are compared to the others. (**b**) 95% confidence interval Tukey test for the number of vascular grafts that reached phase III between animal models. The quantitative data are expressed as the mean ± SD, where * *p* ≤ 0.05, ** *p* ≤ 0.01, *** *p* ≤ 0.001, **** *p* ≤ 0.0001 when compared between models.

Furthermore, the review showed that the proposed critical phases and types of failure are observed in every animal model. However, studies involving smaller animal models are characterized by shorter evaluation times, and therefore they arrive at later phases of integration faster. Additionally, while preseeding cells over grafts before implantation represent an advantage in the initial phases of integration, preventing an instant occlusion and stabilizing the immune response, when the regeneration process reaches the phase of remodeling of the vascular wall, there is no difference on long-term patency between preseeded and non-preseeded grafts. (Figure 4).

According to our analyses, for phase, I failure is generally associated with thrombus formation and foreign body reaction. In contrast, for phase II, failure after the peri-implantation period can be attributed to the interactions of the graft with immune cells and the absence of hemodynamic stabilization. Finally, after overcoming this phase, success in phase III appears related to the ability of TEVG for supporting cell migration and the induction of the remodeling processes in the vascular wall.

To explain the critical points that might unchain events related to failure, we gave each of the phases different possible outcomes. These outcomes were established according to the failure causes reported on in vivo studies for vascular grafts and based on our research group experience. The first two or three outcomes aim to explain the metabolic processes that would lead to a non-regenerative response and/or patency loss due to the incompatibility of the TEVG with the native tissue in hemodynamic conditions. On the other hand, the last outcome would describe the ideal conditions to overcome the phase (i.e., the biochemical and mechanical success signals occurring in a biocompatible implanted vascular graft). The mechanistic explanation of the interactions in the TEVG triad that could elucidate success or failure outcomes are described below. For this review, we will focus on the first phase, in which the first inflammatory responses drive the most critical step, determining the beginning of the vascular wall regeneration.

## 4. Phase I of Vascular Graft Response

### 4.1. Peri-Implantation Period Conditions

The peri-implantation conditions comprise the moments before surgery, during surgery, and the recovery time. During this period, the VG begins the interaction with the vascular microenvironment. Furthermore, the VG is manipulated and sutured and the final geometry is defined.

The microenvironment of the peri-implantation period is one of an altered physiology, with activated signaling cascades from the immune system and new hemodynamics due to the adaptation to the VG geometry as well as any possible structural and integrity change derived from surgery and inflammatory processes. Once a TEVG is implanted, there is a response during the first 24 h oriented towards the control of pathogenic microorganisms, and changes in the vascular permeability to favor cell recruitment and remodeling at the injured site. In this sense, in the first days, the acute inflammatory response provides the cues for cell proliferation, differentiation, and survival factors, as well as growth factors for neovascularization. Furthermore, there is the presence of neutrophils, monocytes, lymphocytes, T cells, and mast cells [67].

From the analysis of the reviewed preclinical studies, we found that on average 16.6% of the implanted TEVGs in different species fail within the peri-implantation period. This has been explained by instant occlusion (related to thrombi) or an exacerbated foreign body reaction. From the specimens unable to continue the pathway to integration, more than 90% of the failure causes were related to thrombus formation.

### 4.2. CASE A: The Vascular Graft Withstands Implantation, but Occlusion Occurs Rapidly

The failure of a VG within the first hours is mainly the result of the development of thrombosis. Thrombosis within the first 24 h can be triggered by the coagulation cascade proteins and consequent platelet adhesion and activation, or by direct platelet activation due to non-physiological shear stress due to the geometry change. As a result, VGs occlude with an uncontrolled thrombus formation and, in the case of TEVGs, the graft’s inability to provide the required oxygen supply and nutrient delivery for a uniform cell distribution and endothelium maturation [68].

#### Coagulation Cascade in Non-Inflammatory Conditions but Response to Contact with Surfaces and Non-Stable Hemodynamic Conditions

The first event after the VG implantation is the protein adsorption, mainly with albumin and fibrinogen due to their higher concentration in blood regarding other proteins, and the protein adsorption mainly depends on the protein properties and biomaterial surfaces [69,70,71]. Fibrinogen adsorption has been linked with thrombogenesis through the interaction with the platelet glycoprotein complex (GPIb-IX-V) and the monocyte activation through the interaction with the Macrophage 1 antigen (Mac1) receptor [72].

For instance, fibrinogen is a small protein that will be located nearer to the vascular wall due to the particle-size-dependent flow distribution in hemodynamic conditions, and its adhesion is also favored by hydrophobic surfaces with positive charges due to the fibrinogen’s D and E domains, negatively charged, as well as several hydrophobic areas that will create electrostatic and hydrophobic interactions. In addition, due to the negative charge of fibrinogen, the interaction with thrombin is also enhanced in its positive charge, thereby promoting thrombogenesis [5].

A previous in silico study from our research group found that under pulsatile flow conditions, fibrinogen adsorption saturation occurs at a time of 0.8 s, while albumin adsorption was found to occur at 1.39 s in hydrophobic surfaces. Similar results have been found in a time-dependent percentage of surface saturation reported by Manzi et al. [73] and the values reported by Mott [74]. For that reason, hydrophobic surface has been correlated with thrombogenesis in PTFE and PET VGs [6] On the other hand, although albumin is also negatively charged, it is mainly hydrophilic. Therefore, a hydrophilic surface is desired in TEVGs to create weak interactions with fibrinogen and increase the albumin adsorption avoiding thrombogenesis. Therefore, different surface functionalization methods have been proposed in the rational design of TEVGs [7].

Likewise, Fröhlich et al. [75] demonstrated an in vivo albumin adsorption on the luminal and external surfaces of VGs as well as several lipoproteins. For instance, glycosylated polar lipids, typical of patients with hyperlipidemia, are prone to adsorb on the luminal surface of the VG, thereby leading to altered shear stress and altered fatty metabolism in inflammatory cells, promoting atherogenesis and contributing to the failure causes of VGs and TEVGs [75].

Furthermore, shear stress has shown to be involved in determining the early fate of the VG because it alters the protein adsorption not only by effect of the flow complexity and the predominance of inertial forces [70,76], but also through the mismatch in the geometry and the mechanical properties at the anastomosis between the graft and the adjacent blood vessel. In some cases, VG implantation might even create branches and curvatures with transition flow patterns correlated to low shear stress values [77]. The decrease in shear stress under such conditions has been reported to promote the unfolding of the von Willebrand factor (vWF) and vitronectin adhesion. Once adsorbed, these proteins undergo conformational changes, allowing the interaction with the GPIb-IX-V and αIIβb3 receptors in platelets or with the Factor 8 (F8) from the coagulation cascade [78].

Upon activation, both GPIb-IX-V and αIIβb3 trigger a signaling pathway responsible for the assembly and activation of proteins related to platelet cytoskeleton remodeling processes such as the Proto-oncogene tyrosine-protein kinase (Src) and the Phospholipase Cγ2 (PLCγ2). Cytoskeleton remodeling leads to adhesion through podosome-like actin nodules [11]. An increased stiffness in biomaterials has also been linked with a thrombogenic potential due to an increased podosome generation. For that reason, the elastic modulus of a TEVG should also be considered to match the properties of the native tissue.

Platelets that have been adhered and become activated will release granules with adenosine diphosphate (ADP) and thromboxane A2 (TXA2), which, in turn, interact with other platelet receptors (e.g., P2Y and TXA2R), enhancing platelet aggregation and autoactivation through the actin remodeling pathways. GPIb-IX-V can also interact with collagen from the exposed anastomosis.

**Figure 5:**
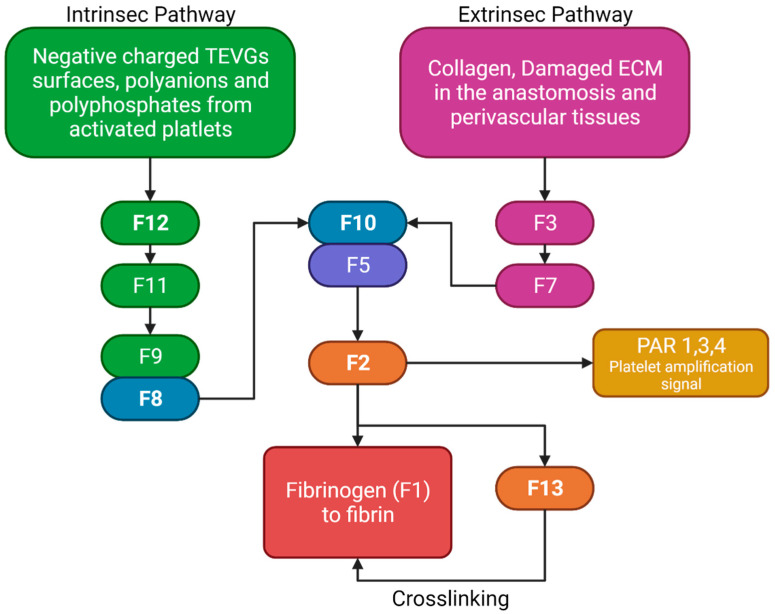
Brief review of the coagulation cascade activation of biomaterials and its different properties. F2 will bind to the protease-activated receptors (PAR) in platelets, causing activation signals for cytoskeleton remodeling. Fibrinogen and F13 will interact for the thrombus stabilization. (Created with BioRender.com, accessed on 7 November 2021).

The two pathways in the coagulation cascade are present in the VG implantation and are responsible for stabilizing clots. Figure 5 shows a brief resume of both ways interacting with VG surfaces. The intrinsic pathway occurs when the kininogen, prekallikrein, and factor 12 (F12) come into contact with polyanions, polyphosphates, or negatively charged surfaces in which they adsorb and become activated. Activated F12 will transform the prekallikrein into kallikrein, and with kininogen it will activate the factor 9 in F9a. In the extrinsic pathway, the damaged cells in the vascular wall will release the tissue factor (F3), that will be binding to F7 and F7a, activating them to form the extrinsic complex. F7 will also be activated due to the thrombin, and small amounts normally circulate in the blood. The F3–F7 complex on the cell membranes will cleave F10 to F10a in the presence of calcium, thereby forming the prothrombinase complex to assemble thrombin. Moreover, F3–F7 also activates F9. In the presence of F4 (Ca^2+^), F8, F3, and platelets, both pathways will transform the factor 10 into F10a, triggering a chain of events leading to factor 2 (F2-thrombin) activation. F2 cleaves the circulating soluble fibrinogen into insoluble fibrin to form a clot network. Active platelets also release α granules containing fibrinogen and coagulation factors F4, F5, and F8, which act as intermediaries in the production of F2 [12]. For instance, it has been reported that 4-mm PTFE grafts show high levels of F12a and F2 [13].

Nevertheless, although some TEVGs have reported the use of inhibitors of the F10a or F2a (e.g., Heparin), which have also been used in commercial grafts, there is a gap in the literature that explains the specific behavior of different biomaterials with the coagulation cascade in the hemodynamic context of a VG [8]. Collagen-based TEVGs are expected to have a higher impact on the extrinsic pathways, while negatively charged polymers or surfaces will preferentially activate the intrinsic pathway. For example, this would be true for polymers such as polylactic aid, in which a negative charge on the surface will be obtained due to the lactic acid resulting from degradation, thereby generating a constant stimulus for the intrinsic pathway activation [9].

On the other hand, a low shear stress disturbs the physiological velocity profile due to its associated transitional flows on sharp geometrical discontinuities (such as anastomotic sites) in vessels inducing the red blood cells’ (RBCs) arrangement on the VG periphery and generating an electrostatic interaction [14,15]. The RBCs’ surface proteins, such as integrin, laminin, and fibronectin, have been shown to adhere to the VG surface through unspecific interactions. Adhered RBCs release adenosine triphosphate (ATP), which is subsequently converted to ADP and TXA2 [79]. If the material promotes hemolysis, the released hemoglobin scavenges nitric oxide (NO), a platelet inhibitor that blocks the activation of GPIb-IX-V. Both mechanisms have been reported to promote thrombogenesis [80]. For instance, PET VGs show an enhanced interaction with platelets and RBCs, mainly due to their high permeability [13].

In summary, the surgical procedure, the material properties, and the altered hemodynamic conditions can cause platelet aggregation and activation and a fast auto-amplification signaling via the coagulation cascade. The ultimate result is a fast activation of thrombogenesis mechanisms that leads to an instant occlusion of the VG.

### 4.3. CASE B: The Vascular Graft Fails Immediately due to Complications Related to a Strong Foreign Body Response

The failure of a VG within the first days is mainly a result of uncontrolled acute immune responses. The inflammatory response in a VG occurs by a combination of the impact of the injury, the material properties, and the hemodynamic response.

#### 4.3.1. Thrombogenesis-Mediated Inflammation

**Figure 6:**
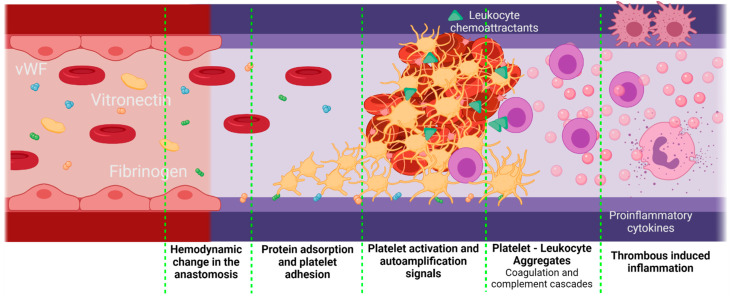
Proposed scheme for platelet–leukocyte aggregates generated in vascular grafts. Platelet–leukocyte aggregates might be favored if there is a significant damage of the adjacent tissues during the implantation, as well as an inadequate hemodynamic behavior of the VG with a thrombogenic surface. In this case, the first step is protein adsorption. Due to the surgery, the F3 coagulation factor will be released, activating the extrinsic coagulation pathway. On the other hand, kininogen, prekallikrein, and factor 12 (F12) will attach to negatively charged surfaces, activating the intrinsic pathway and, finally, under low shear stress conditions, vWF is unfolded, thereby interacting with the platelet receptors. Active platelets adhere to the surface of the vascular graft and promote coagulation by autoamplification mechanisms. Active platelets also release leukocyte chemoattractants and leukocyte-binding proteins such as the P-selectin. The leukocytes adhered through the P-selectin glycoprotein ligand-1 will release a series of pro-inflammatory cytokines, which enhances the inflammatory response even further (Created with BioRender.com, accessed on 7 November 2021).

Inflammation can be influenced by thrombus formation. If a thrombus has been formed, the activated platelets release granules containing fibrinogen, coagulation factors that activate and amplify the coagulation cascade, and molecules responsible for the complement cascade activation. As a result, platelets initiate not only thrombogenic events but also inflammatory responses [81]. A crosstalk between the complement cascade and the platelets enhances the procoagulant responses mediated by inflammation. The activated platelets release P-Selectin, chemokine ligand 5 (CCL5), and chemokine ligand 5 (CXCL4), which are leukocyte chemoattractants. The presence of different GP receptors in platelets favor the interaction with the Mac 1 receptors on the leukocytes, thereby creating platelet–leukocyte aggregates (PLA) on the VG. Figure 6 shows a brief representation of the PLA generation on VGs. The PLA crosstalk increases the inflammatory and thrombogenic response by releasing factors of the coagulation and complement cascades [82]. Although this specific event represents a gap in the literature on VGs, it is recognized that PLA aggregates are very common in the development of cardiovascular diseases in which the alteration of the endothelium will promote this cascade of events, in which patients have shown an increase in PLA markers such as P-Selectin from the platelets and the P-selectin glycoprotein ligand-1 from the immune cells. For example, Esposito et al. showed that 41% of patients undergoing peripheral vascular surgery with synthetic vascular grafts, although non immediately, presented graft occlusion characterized by an increase in the monocyte–platelet and neutrophil–platelet conjugates [19].

#### 4.3.2. Complement Cascade

The complement cascade is a well-known protein-mediated system that interacts through enzymatic mechanisms to attract immune cells to the damaged tissue. Three different pathways activate the complement system. The classical pathway where C1q proteins recognize fragments of damaged cells, the lectin pathway where mannose-binding lectin (MBL) binds to sugars on the damaged cells (or to bacteria if there is an infection), and the last pathway involve small amounts of C3 protein that have been hydrolyzed to bind to other complexes. All the pathways converge in the activation of the C3 convertase, which cleaves C3 to generate C3a and C3b. While C3 is a direct chemoattractant of leukocytes, C3b joins to a different complex to generate C5a and C5b. C5b promotes the lysis of damaged cells or bacteria, but C5a is a leukocyte chemoattractant with potent platelet activator capabilities, through interactions with leukocyte and platelet receptors. Accordingly, the PLA complex is mainly responsible for the activation of the alternative pathway with the platelets to induce C3 hydrolysis, followed by the C5 auto-activation [83]. Recent reports show that VGs exhibit a low platelet aggregation and a reduction in the amount of C3a compared non-endothelialized controls and PTFE [84].

Protein adsorption on the VG’s surface regulates the interaction between the cells and the biomaterial surface, thereby largely inhibiting cell adhesion. This adsorption processes occur during the early stages after VG implantation due to their affinity to specific moieties on the surface and their surfactant behavior. Hydrophobic materials such as polyurethanes (PU) and PET easily attract and bind host proteins from the plasma and interstitial fluid. The surfactant power of certain proteins is the result of their large size, the presence of a wide array of side chain function groups, and a partial protein unfolding to obtain stability under physicochemical conditions [85]. These properties led to noncovalent interactions between the proteins and the biomaterial surface, including electrostatic, van der Waals, and entropic interactions. Additionally, stronger interactions might be induced by the denaturation of proteins on the hydrophobic surfaces, and unique hydrophobic interactions, which generally last for the first weeks and prevent cell adhesion [69,86].

C3a inhibition has also been shown to decrease vein graft atherosclerosis in mice models due to a decreased leukocyte attraction [87]. Additionally, increased levels of C5b have been found in PET VG compared to polymeric TEVGs [13]. These findings confirm not only the participation in the activation of the complement cascade in VGs’ failure but also the regulatory role of the endothelial cells (ECs) in thrombogenic and proinflammatory responses and the importance of endothelialization.

#### 4.3.3. Cytokines and Degradation Products

Regarding the specific inflammatory process and cell recruitment after the VG implantation surgery, it has been reported that some of the degradation proteins from the ECM, such as hyaluronic acid, are released and are also known as damage-associated molecular patterns (DAMPs). These molecules are recognized by the toll-like receptors (TLR) or by the triggering receptor expressed on myeloid cells 1 (TREM-1), causing monocyte attachment and activation. Monocyte activation promotes the release of the macrophage colony-stimulating factor (M-CSF) and monocytes chemoattractant protein 1 (MCP1), promoting the leukocyte colonization on the lumen of the VG [28]. On the other hand, during the first 24 h post implantation, neutrophils from the perivascular tissue have been reported to infiltrate into the graft wall and amplify the signal, thereby leading to the release of MCP1 for the monocyte chemiotaxis [88]. Such an effect has been consistently observed in VG, where the number of monocytes is correlated with the number of present neutrophils [15].

In animal models, it has been reported that, on the first days, the predominant cells on the VG walls are neutrophils, but there are also infiltrated monocytes that differentiate towards macrophages [89,90]. It is known that both neutrophils and macrophages are responsible for the biomaterial degradation through the release of matrix metaloproteinases (MMPs) and reactive oxygen species (ROS). MMPs are themselves adhesive molecules to other monocytes. In saphenous vein grafts, in vitro experiments have shown an increase in the MMPs 2 and 9 in a ROS-dependent manner [91]. ROS are free radicals intended to break chemical bonds apart in the material to finally degrade it. However, ROS levels increase if the material resists degradation. Elevated ROS act as a second messenger interacting with redox-sensitive proteins, including receptors and transcriptional factors. In leukocytes, ROS induce the expression of intercellular adhesion molecules (ICAM-1), promoting the leukocyte-binding and -infiltration. The inflammatory response is further enhanced by the increased concentrations of M-CSF and MCP1 [92].

The transitional flow and low shear stress at the anastomosis have also been shown to increase the ROS levels. Under such conditions, the expression of proinflamatory proteins such as the tumor necrosis factor-α-mediated factor (TNF-α) is exacerbated by the activation of the JNK (c-Jun N-terminal kinase)/p38 kinase pathway [93]. TNF-α is well known to act as a signaling molecule recognized by the TNF receptor on the surface of leukocytes to activate proinflammatory and proliferative pathways.

Even though allergenic responses have only been reported in a few VG cases [94,95], activated mast cells have been observed in the perivascular tissue of TEVGs but fail to infiltrate into the graft itself [96]. Mast cells are known to release granules with histamine, chemokines and adhesive molecules to promote leukocyte infiltration [96]. In this sense, a local shock-like inflammatory response might be rapidly auto-amplified, thereby causing a disproportionate immune reaction to induce blood vessel contraction. This event, added to the previous thrombus formation, marks the early patency loss of VGs due to an excessive inflammatory response. For example, it has been reported that patients that undergo endovascular aneurysm repair with vascular grafts (PET) have a higher risk (14 to 60% of the total of patients) of developing a dangerous acute-phase systemic inflammatory response [97].

#### 4.3.4. Bacterial Infection

Although rare, infection is a possible cause of failure upon graft implantation. The most common pathogens during VG implantation are staphylococcal species and *E.-coli*-abundant patient skin. After implantation, there are two possible infection peaks. The first one can occur during the early postoperative period, in which the infection is the result of contamination in surgery, while the second one occurs later on because of bacteremia seeding on the graft. This second type of infection will cause the instability of the immune response and does not allow for a proper microenvironment for regeneration. This is especially important because infections coming from bacteria in the graft will start showing symptoms and affecting the systemic response from the body when the proliferation of cells within the graft should start to promote vascular regeneration. In consequence, the infection resolution will compete with the regeneration of the vascular wall.

Infections happen in 0.2 to 5% of intracavitary grafts (in the abdomen and thorax) and in 6% of the cases in extracavitary grafts [36]. When infection does happen, the usual protocol is to withdraw the graft, because mortality rates range from 24 to 75% in the intracavitary cases and in about 17% in the extracavitary ones. The most common site for post-operative graft infection is the groin, where the infection can even result in limb loss [98]. When a graft is removed, bypasses might be needed. For instance, in the case of arteriovenous grafts, removing the graft will delay the starting point for dialysis, which is critical in patients with chronic kidney disease. The starting point is of special importance because bypass maturation periods oscillate from 33 to 54 days [3].

The probability of infection is related to the type of graft that is being implanted, as it depends on surface characteristics such as hydrophobicity, electric charge, functional groups, micro-pattering, roughness, and porosity of the material [99]. PTFE grafts are the least susceptible to infection, while grafts from biological origin and PET are respectively 5 and 50 times more prone to infection [99]. VG endothelialization has proven to reduce bacterial adhesion in animal models [100], and therefore tissue-engineered VGs represent an alternative to prevent infections.

### 4.4. CASE C: The Graft Is Successfully Implanted at the Intervention Site and the Inflammatory-Remodeling Processes Begin

It has been reported that if the TEVG successfully overcome the peri-implantation period, hematopoietic stem cells (HSCs) and already differentiated monocytes—macrophages—infiltrate into the material during the first 2 to 10 days. This infiltration occurs as a response of the MCP1 activity from monocytes interacting with DAMPs [28] and from mast cells in the periphery [96]. Another reported mechanism is the release of the macrophage inflammatory protein 1-alpha (MIP-1) produced by natural killer cells (NK) that infiltrate the vascular wall after the first days of implantation [101].

#### 4.4.1. Macrophage’s Polarization

In physiological conditions, in response to local stimuli during the wound healing process, macrophages can modulate their polarization into M1 and M2 phenotypes. While M1 phenotype macrophages are associated to the proinflammatory pathways (inflammation, fibrosis, and material encapsulation), the M2 phenotype macrophages are associated with an anti-inflammatory response that triggers cellular recruitment, differentiation and proliferation, angiogenesis, and the degradation of bioresorbable materials [42,102].

**Figure 7:**
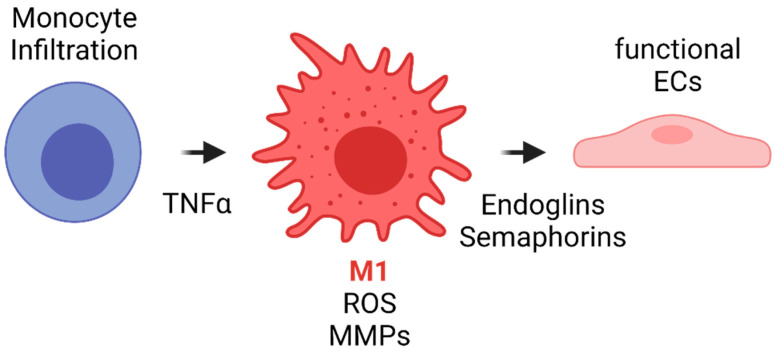
Proposed scheme for the acute inflammation role in TEVG’s endothelialization. Monocyte infiltration occurs during the first days after implantation. The inflammatory response due to different cytokines and TNFα induces monocyte differentiation towards M1 type macrophages. M1 macrophages will initiate the biomaterial degradation process through MMP release and by ROS generation and auto-amplification signals. During the first weeks, M1 will release endoglins and semaphorins, both of which are chemoattractants for endothelial progenitors, to begin the endothelialization in the lumen and the angiogenesis in the vascular wall (Created with BioRender.com, accessed on 7 November 2021).

The literature has shown that during the first weeks after implantation, macrophages within the VG have been reported to develop a M1 phenotype due to the proinflammatory cytokines released during previous events such as interleukin 1 (IL-1). Such phenotype is maintained by the TLR and TNF-α signaling. Although this event has not been studied in detail in vascular grafts, it is known that M1 macrophages have different functions. Firstly, M1 appear to be related to an increase in the leukocyte recruitment by the release of MCP1 and other proinflammatory cytokines. Secondly, M1 macrophages exhibit a high phagocytic activity toward material degradation via matrix metalloproteinases (MMPs) and ROS release. Finally, even in the situations of exacerbated inflammatory responses responsible for VG failure, M1 have been shown to release endoglins and semaphorins that are chemoattractant for endothelial cells. In fact, one of the sources of cells for endothelial lining in VG comes from transmural capillary infiltration through porous grafts from a granulated tissue capsule [103]. Figure 7 shows a proposed scheme of the role of acute inflammation in TEVG’s endothelialization.

Other types of inflammatory cells have also been shown to be attracted by the perivascular tissue in VGs, including dendritic cells and lymphocytes T and B [88]. However, they have not been correlated with vascular graft failure, which suggests that an innate immunity response plays a more significant role than humoral immunity in the performance of vascular grafts [101].

After the first weeks, the cell response to the material and the hemodynamic conditions are the main determinants in chronic inflammation or regeneration. Chronic inflammatory outcomes typically induce intimal hyperplasia and atherosclerosis. However, regenerative pathways promote reendothelialization, which is one of the most important steps towards recovering the function of the replaced artery. We will review these two possible outcomes separately.

#### 4.4.2. Inflammation Resolution

After the first weeks, the massive death of neutrophils and macrophages has been reported to occur in VG, having two main effects. First, the release of their inner contents activates signaling cascades from the other leukocytes, and ECs responds to promote the production of IL-4 and IL-13. Second, the interaction of macrophages with apoptotic cells inhibits the NADPH oxidase and, consequently, leads to a decrease in ROS production by the macrophages. However, this mechanism has been shown to improve the phagosomes’ proteolytic activity. Under these conditions, macrophages are no longer able to secrete molecules to degrade the material, but acquire a phenotype that allows for the phagocytosis of the necrotic tissue. This switching process usually occurs within the first month after implantation [104].

**Figure 8:**
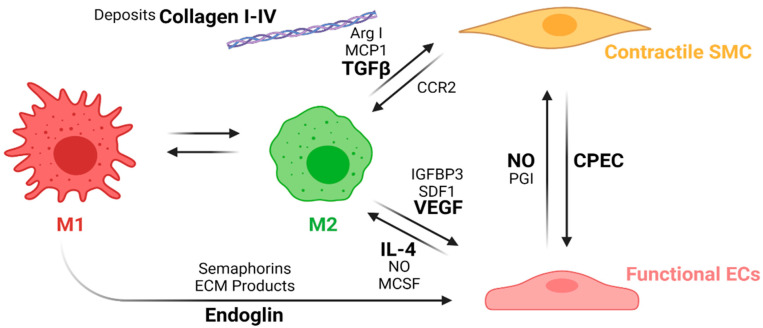
Macrophage polarization effects on the cell phenotypes in the regeneration process of the vascular wall. During the first weeks, M1 macrophages stimulates EC attachment and proliferation through the release of endoglins, semaphorins, and the ECM products during the phagocytosis. After the first weeks, inflammation resolution begins with macrophage populations switching towards an M2 phenotype due to the IL-4 and MCSF released by functional ECs as well as an increase in the NO levels produced by the ECs. M2 macrophages also induce vascular wall remodeling by increasing ECs recruitment and maturation through the release of VEGF, IGFBP3, and SDF1. M2 also induce the SMCs’ expression profile towards a contractile phenotype through the release of TGFβ, ArgI, and MCP1, while contractile SMCs help the maintenance of the M2 population with the interaction of the CCR2. The interplay between mature ECs and contractile SMCs contributes to maintaining the vascular wall tone and patency (Created with BioRender.com, accessed on 7 November 2021).

This process is known as macrophage polarization, where macrophages develop an alternative activated phenotype or M2, which is thought to mediate the remodeling of the new tissue in four different ways [68]. First, M2 release growth factors such as the transforming growth factor beta (TGFβ) and other molecules, such as Arginase I (Arg I) and MCP-1, which induce the migration and proliferation of SMCs. The interaction between M2 and SMCs through the MCP1 and its receptor (CCR2) maintains the M2 state. Second, M2 release the vascular endothelial growth factor (VEGF), the insulin-like growth factor binding protein (IGFBP-3), and the stromal cell derived factor 1 (SDF1) to induce endothelialization. Mature ECs maintain the M2 state through the NO activity and the release of IL-4. Third, M2 release anti-inflammatory cytokines such as IL-10. Fourth, extracellular matrix components are produced, including collagen type I and IV, tropoelastin, fibronectin, and glycosaminoglycans [96]. Once the endothelium is formed, the NO and the PGI induce the expression of the functional phenotype of SMCs (contractile). Contractile SMCs maintain the expression of cyclopentenyl cytosine (CPEC), which inhibits SMC proliferation but promotes EC proliferation. Figure 8 shows a proposed scheme of the macrophage polarization effects on the cell phenotype development during the regeneration process of the vascular wall.

Although many authors have designed TEVGs with less porous outer layers to avoid leakage or to maintain the mechanical properties, it is necessary to understand that the cells that will remodel the TEVG have two sources: the blood and the peripheral tissues. However, there is more recent evidence that the cells in the vascular wall come mainly from the peripheral tissue, and this will only happen if the vascular wall is surrounded by granular tissue [68]. For that reason, the TEVG microstructure affects cell infiltration and subsequent remodeling, and one of the main features of a TEVG should be the porosity of the conduit. This will determine its capacity to support the microcapillary formation through the vascular wall to allow cell migration from the perivascular tissue towards the graft lumen [43]. It has been found that VG with pores ranging from 30 to 90 µm are more likely to allow cell infiltration and maintain longer patency rates [68]. On the contrary, either capillary growth will be impaired or the infiltration of fibrous tissue will be enhanced.

Furthermore, to overcome the inflammatory responses and start a physiological regeneration process of the vascular wall, the microenvironment of the VG should be optimized not only to allow for cell infiltration, but also to induce reendothelialization. This means that the structure of the VG needs to be tailored for cell adhesion and migration through the balance of inflammatory cues that promote progenitor endothelial cells towards a functional endothelium, due to its modulatory roles over macrophages and SMCs.

**Figure 9:**
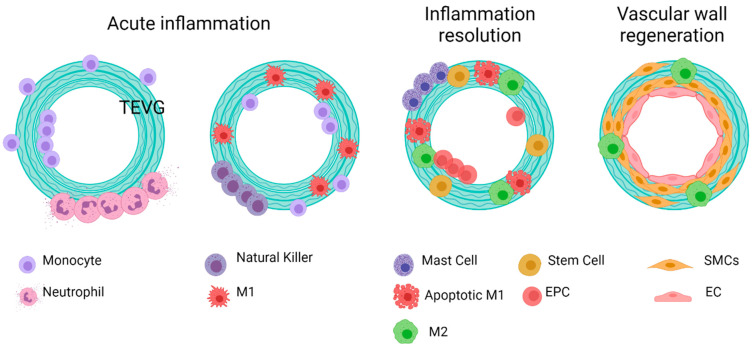
General idealized and summarized cell biomaterial interaction for the reendothelialization mechanism as a result of immune response activation over TEVGs. On the desired outcome, during the first stages after the implantation of regenerative vascular grafts, neutrophils and monocytes adhere to the inner and outer surfaces. The chemoattractant responses of these cells induce the infiltration of different kinds of cells, including hematopoietic stem cells. The acute inflammation stages induce the attachment of endothelial precursors. After one month, infiltrated cells on the vascular wall will differentiate towards functional smooth muscle cells and fibroblasts, and the adhered endothelial cells on the lumen will maturate. Therefore, a healthy endothelium will provide the required homeostasis for maintaining vascular graft patency (Created with BioRender.com, accessed on 7 November 2021).

**Figure 10:**
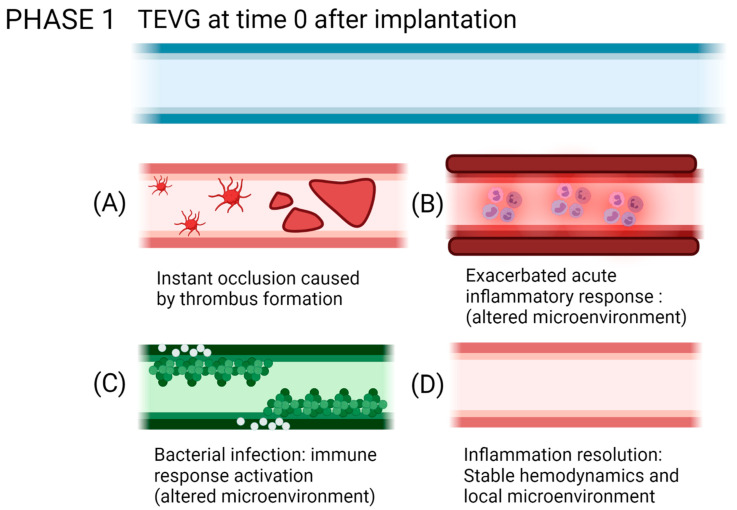
Possible outcomes of the interaction of TEVGs with the native microenvironment if the failure mechanisms are activated within the early stages of integration. (**A**) Instant thrombus formation causes a significant reduction of the lumen of the graft, (**B**) while an exacerbated inflammatory state reduces the possibilities to initiate a regenerative pathway. (**C**) If infection occurs, the pathogens will repopulate the graft instead of the native cells. (**D**) If after acute inflammation, the microenvironment stabilizes; the graft becomes a suitable substrate for tissue remodeling (created with BioRender.com, accessed on 7 November 2021).

Reendothelialization, which is the formation of a lining of endothelial cells over the surface of a VG, is a process that is still under study, and this step is critical to provide homeostasis in the vascular wall, given the multiple endothelium function in controlling the vascular wall permeability, compliance, and inflammation. Further details on vascular wall regeneration and endothelial formation can be studied separately. Figure 9 and Figure 10 shows a brief summary of the cell-biomaterial interaction required for the reendothelialization mechanism as a result of immune response activation over TEVGs, as well as the possible outcomes of the interaction of TEVGs with the native microenvironment if the failure mechanisms are activated within the early stages of integration.

## 5. Conclusions

- Studies involving smaller animal models are characterized by shorter evaluation times, and therefore they arrive more quickly at later phases of integration. We found statistically significant differences in the percentages of success survival within smaller and larger animal models, as dog–rat and pig–rat. Vascular grafts that do not overcome the first phases of regeneration mainly fail due to thrombogenic events or bacterial infection in different ways. First, either the biomaterial is non-hemocompatible, the surface promotes the activation of the coagulation cascade and the platelet activation, or the released molecules from hemolyzed red blood cells activate the platelets. Second, altered hemodynamics will cause the von Willebrand factor (vWF) and vitronectin adhesion, causing platelet activation. Third, an exacerbated inflammatory response will generate platelet–leukocyte aggregates. Fourth, although rare, infections are also dependent on the biomaterial properties and might be life-threatening.
- Once the first phase of regeneration is crossed, there are two different events during the acute inflammatory response in TEVGs, considering that the cells’ sources for vascular wall regeneration can be the blood flow and the peripheral tissues. First, different kinds of cells, including monocytes and stem cells, infiltrate the vascular wall from the granulation tissue in the periphery, and regeneration will be allowed depending on their response and interaction,. Second, type-1 macrophages secrete integrins and semaphorins, which are chemoattractants for endothelial progenitors from the blood flow that will adhere to the lumen surface of the vascular graft and begin reendothelialization.
- If the regenerative pathway is activated, macrophage polarization occurs within the vascular wall, and type-2 macrophages release growth factors and cytokines that induce cell differentiation towards contractile smooth muscle cells and functional endothelial cells.

## Figures and Tables

**Table 1:** Vascular graft features of the analyzed pre-clinical animal models.

	Caprine	Swine	Dog	Rabbit	Rat
Av. Diameter (mm)	5	4.3	4.4	2.8	1.7
Av. Length (mm)	47	50	45	21	8710
Subjects	2 to 20	3 to 10	1 to 20	3 to 18	4 to 24
Following time	1 to 9 months	7 days to 5 months	1 to 12 months	1 month to 3 years	1 to 12 months
Number of studies	19	18	36	23	23

**Table 2:** Summary of pre-clinical animal models analyzed for this study.

	Author, Year	Ref.	Cells Seeded	Luminal Cell Type	External Cell Type	Length (mm)	Diameter (mm)	Scaffold Type	Localization
**Sheep**	Tillman, 2012	[21]	Yes	PB-EC	None	60	5.0	Decellularized Artery	Carotid Artery AV Shunt
	Kaushal, 2001	[22]	Yes	PB-EC	None	45	4.0	Decellularized Artery	Carotid Artery
	Cho, 2005	[23]	Yes	BMMNC-EC	BMNC-SMC	40	3.0	Decellularized Artery	Carotid Artery
	Koenneker, 2010	[24]	Yes	PB-EC	None	75	5.0	Decellularized Artery	Carotid Artery AV Shunt
	Neff, 2011	[25]	Yes	PB-EC	Vessel SMC	60	5.0	Decellularized Artery	Carotid Artery
	Koch, 2010	[26]	Yes	None	Vessel SMC	45	5.0	Hybrid (Firbin-PLA)	Carotid Artery
	Ju, 2017	[27]	Yes	PB-EC	Vessel SMC	50	4.8	Hybrid (Collagen-PCL)	Carotid Artery
	Fukunishi, 2017	[28]	No	None	None	15	12.0	Synthetic (PGA-PCL)	Inferior vena cava
	Row, 2015	[29]	Yes	EC	SMC	45	5.0	Natural (SIS- Fibrin)	Carotid Artery
	Aper, 2016	[30]	Yes	PB-EC	PB-SMC	90	5.6	Natural (Fibrin + Coagulation Factor XIII)	Carotid Artery
	Swartz, 2005	[31]	Yes	Vessel-EC	Vessel-SMC	12	5.0	Natural (Firin-Thrombin)	Yugular Vein
	Meier, 2014	[32]	Yes	PB-EC	None	25	4.0	Natural (Fibrin gel)	Femoral Artery
	Ramesh, 2013	[33]	No	None	None	130	4.0	Decellularized Vein	Carotid Artery
	Aussel, 2017	[34]	No	None	None	35	6.0	Natural (Chitosan)	Carotid Artery
	Weber, 2018	[35]	No	None	None	10	4.5	Natural (Nanocellulose)	Carotid Artery
**Goat**	Turner, 2006	[36]	Yes	Vessel-EC	None	45	4.5	Hybrid (PU + cells)	Carotid Artery
	Kim, 2007	[37]	No	None	None	40	4.5	Decellularized Artery	Carotid Artery
**Rabbit**	Zheng, 2012 *	[38]	No	None	None	15	2.2	Synthetic (PCL)	Carotid Artery
	Cutiongco, 2016 *	[39]	No	None	None	Not reported	0.9–1	Synthetic (PVA)	Femoral Artery
	Zhang, 2017 *	[40]	No	None	None	30	4.0	Synthetic (ePTFE)	Abdominal Aorta
	Wang, 2016 *	[41]	No	None	None	10	2.2	Synthetic (PCL)	Carotid Artery
	Wise, 2011 *	[42]	No	None	None	20	2.8	Hybrid (PCL—Elastin)	Carotid Artery
	Tillman, 2009 *	[43]	No	None	None	40	5.0	Hybrid (PCL—collagen)	Aortoiliac Bypass
	Evans, 2015 *	[44]	No	None	None	30–40	2.0	Autologous vein	Yugular Vein
	Zhu, 2012 *	[45]	Yes	HOB	None	5	3	Synthetic (PCL)	Femoral Artery
	Ishii, 2008 *	[46]	No	None	None	24	3.6	Hybrid (PU—collagen—Hyaluronic acid)	Abdominal Aorta
	Kajbafzadeh, 2019 *	[47]	No	None	None	Not reported	Not reported	Decellularized Artery	Femoral Arteries
	Zhao, 2019 *	[48]	No	None	None	20	2	Synthetic (PLCL)	Carotid Artery
	Bai, 2019 *	[49]	No	None	None	20	2	Synthetic (PS)	Carotid Artery
	Bai, 2018 *	[49]	No	None	None	20	2	Synthetic (PS)	Carotid Artery
**Pig**	Koens, 2015	[50]	No	None	None	35	4	Natural (elastin-collagen)	Iliac Artery
	Rotmans, 2005	[51]	No	None	None	70	5	Synthetic (PTFE+ CD34 coating)	Carotid Artery AV Shunt
	Rothuizen, 2016	[52]	No	None	None	40	4.2	Fibrotic capsule tube + PCL	Carotid Artery
	Mahara, 2015	[53]	No	None	None	250	3	Decellularized Artery	Femoral Artery
	Sánchez-Palencia, 2018 *	[54]	No	None	None	17	4.5	Decellularized SIS	Carotid Artery
	Dahan, 2017	[55]	No	None	None	45	4.0	Decellularized Artery	Carotid Artery
	Valencia Rivero, 2017	[56]	No	None	None	150	4.0	Decellularized SIS	Carotid Artery AV Shunt
	Zavan, 2008	[57]	No	None	None	50	4.0	Natural (Hyaluronan)	Carotid Artery
	Wippermann, 2009	[58]	No	None	None	10	3.4	Natural (Cellulose)	Carotid Artery
	Hinds, 2006	[59]	No	None	None	40	4.3	Natural (Ellastin)	Carotid Artery
	Pellegata, 2015	[60]	Yes	hIAECS	None	50	6.0	Decellularized Artery	Iliac Artery
**Dog**	Arts, 2002	[61]	Yes	MVEC	None	50	4.0	Synthetic (ePTFE)	Carotid Artery
	Xie, 2010	[62]	No	None	None	50	6.0	Poly (carbonate urethane) (PCU) filaments	Infra-renal aorta
	Yokota, 2008	[63]	No	None	None	30	4.0	Collagen microsponge with a biodegradable woven polyglycolic acid (core) and poly-L-lactic acid (sheath) fibers.	Carotid Artery
	Zhou, 2009 *	[64]	No	None	None	45	3.0	Decellularized/hybrid	Carotid Artery
	Zhou, 2012	[65]	Yes	PBMC-EC	None	45	3.0	Decellularized Artery	Carotid Artery
	Zhou, 2014 *	[66]	No	None	None	45	3.0	Hybrid chitosan/poly(e-caprolactone) (CS/PCL) nanofibers	Carotid Artery

* Additional studies included different from the reported by Skovrind et al [19].

## Data Availability

The data presented in this study are available on request from the corresponding author. The data are not publicly available due to confidentially.
